# Periplaneta Americana extract inhibits osteoclastic differentiation in vitro

**DOI:** 10.1111/cpr.13341

**Published:** 2022-10-09

**Authors:** Bin Zhao, Yuning Zhang, Jie Xu, Yuyu Li, Quan Yuan, Chenchen Zhou

**Affiliations:** ^1^ State Key Laboratory of Oral Diseases, National Clinical Research Centre for Oral Disease, West China Hospital of Stomatology Sichuan University Chengdu China; ^2^ Department of Oral Implantology, West China Hospital of Stomatology Sichuan University Chengdu China; ^3^ Department of Pediatric Dentistry, West China Hospital of Stomatology Sichuan University Chengdu China

## Abstract

**Objectives:**

Periplaneta americana extract (PAE) is proven to be promising in treating fever, wound healing, liver fibrosis, and cardiovascular disease. However, the role of PAE in skeletal disorders remains unclear. This study investigated whether PAE regulates osteoclastic differentiation in vitro via the culture using RAW264.7 cells and bone marrow derived macrophages (BMDMs).

**Materials and Methods:**

RAW264.7 cells and BMDMs were cultured and induced for osteoclastic differentiation supplementing with different concentrations of PAE (0, 0.1, 1, and 10 mg/mL). Cell counting kit‐8 (CCK‐8) assay was used to detect the cytotoxicity and cell proliferation. TRAP staining, actin ring staining, real‐time quantitative PCR (RT‐qPCR), and bone resorption activity test were performed to detect osteoclastic differentiation. RT‐qPCR and enzyme‐linked immunosorbent assay (ELISA) were conducted to assay the expression and secretion of inflammatory factors. RNA sequencing (RNA‐seq) and western blot analysis were carried out to uncover the underlying mechanism.

**Results:**

CCK‐8 results showed that 10 mg/mL and a lower concentration of PAE did not affect cell proliferation. RT‐qPCR analysis verified that PAE down‐regulated the osteoclastic genes *Nfatc1*, *Ctsk*, and *Acp5* in macrophages. Moreover, PAE restrained the differentiation, formation, and function of osteoclasts. Besides, RT‐qPCR and ELISA assays showed that PAE decreased inflammatory genes expression and reduced the secretion of inflammatory factors, including IL1β, IL6, and TNFα. Subsequent RNA‐seq analysis identified possible genes and signaling pathways of PAE‐mediated osteoclastogenesis suppression.

**Conclusions:**

Our study indicates that PAE has inhibitive effects on osteoclastogenesis and may be a potential therapeutic alternative for bone diseases.

## Graphical abstract

Periplaneta americana extract (PAE), the animal medicine material extracted from the insects Periplaneta americana, is proven to possess a variety of pharmacological functions. However, the role of PAE in skeletal disorders remains unclear. In this study, we found that PAE decreased osteoclast genes expression *Nfatc1*, *Ctsk*, and *Acp5* in macrophages. Besides, PAE restrained the differentiation, formation, and function of osteoclasts. Moreover, PAE suppressed the LPS‐induced inflammation. Subsequent RNA‐seq analysis identified the signaling pathways of PAE‐mediated osteoclastogenesis suppression. Our study indicated that PAE has inhibitive effects on osteoclastic differentiation and may be a potential therapeutic Chinese medicine for bone diseases.

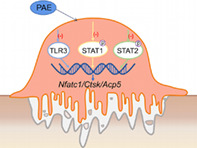

## 1 INTRODUCTION

Osteoporosis is a systemic disease associated with decreased bone density and mass and the destruction of bone microstructure. Physiologically, bone tissue undergoes continuous remodelling through the coordination of bone‐resorbing by osteoclasts and bone‐forming by osteoblasts.^1^ As the balance of bone absorption and bone regeneration is destroyed, the bone absorption of osteoclasts is stronger than the bone reconstruction of osteoblasts, resulting in increased bone fragility, uncomplicated fracture, pain, and a series of symptoms.^2^ Under this circumstance, it is crucial to effectively inhibit osteoclastic differentiation and function.

Osteoclasts, derived from macrophage lineage, are the main cells responsible for degrading the bone matrix. When exposed to cytokines including macrophage colony‐stimulating factor (MCSF) and receptor activator of nuclear factor‐Kβ ligand (RANKL), mononuclear progenitors differentiate into multinucleated giant osteoclasts.^3^ Osteoclastic differentiation is a complex and exquisitely regulated process that requires a series of molecular factors and signaling pathways.^4^, ^5^ Based on the mechanism, therapeutic approaches to inhibit osteoclastic differentiation have been developed. Osteoclastogenic inhibitors could be derived from chemical, biological, or natural strategies.^6^ However, the side effects and lack of long‐term effects of the existing therapies have raised global concern. For example, the chemical‐based agent bisphosphonates can inhibit osteoclast activity but may increase the risk of femur fracture, jaw osteonecrosis, or hypocalcaemia.^7^ Moreover, biological‐based oestrogen has been reported to carry a higher risk of breast cancer and cardiovascular disorders.^8^ It is still challenging to develop osteoclastogenic inhibitors with fewer side effects and prolonged efficacy.

Traditional Chinese medicine has been used to prevent and treat diseases for centuries with fewer side effects. With the increasing research on traditional Chinese medicine, numerous studies have shown that the effective ingredients of traditional Chinese medicine have the potential effect on treating osteoporosis, such as flavonoids and saponins.^9^ Total flavonoids of Epimedium inhibit osteoclastic differentiation and bone resorption by down‐regulating the expression of RANKL.^10^ A traditional Chinese medicine, Fenugreek steroidal saponins, could target CSF‐1R to inhibit bone resorption caused by osteoclasts.^11^ Periplaneta Americana is an insect of Insecta, winged subclass, Blattella, Blattellidae, and Periplaneta.^12^ It is commonly known as a “cockroach” and has strong vitality. As a traditional Chinese medicine, it was first published in Shennong's classic of Materia Medica. Recently, the modern preparation of PAE, Kangfuxin solution, has been widely applied for the therapy of wound healing, gastrointestinal ulcer, and hepatic fibrosis.^13^, ^14^, ^15^ Moreover, the antibacterial, antiviral, and antitumor functions of PAE were also assessed.^16^, ^17^, ^18^ Nevertheless, the potential of PAE in suppressing osteoclastic differentiation and the underlying mechanisms are still elusive. In this study, we first investigated the inhibitive effects of PAE on osteoclastogenesis using both macrophage cell line RAW264.7 and BMDMs. We then screened the possible mechanisms through RNA sequencing. This study aims to show that PAE is a promising agent for mediating osteoclastic bone resorption.

## 2 MATERIALS AND METHODS

### 2.1 Cell culture and differentiation

Bone marrow macrophages from femur and tibia of wild C57BL/6 mice were isolated in a sterile ultra‐clean table, placed in a diameter of 10‐cm dish, and treated with α‐MEM (Gibco) medium, containing 10% fetal bovine serum and 25 ng/mL MCSF (R & D systems). The isolated cells were cultured in 37°C and 5% CO_2_ incubator for 5 days, and the solution was changed every 2 days. Five days later, the adherent cells induced by MCSF were BMDMs. RAW264.7 cells were purchased from Cyagen company, and the culture medium was DMEM (Gibco) containing 10% fetal bovine serum (Gibco). To induce osteoclastic differentiation, 1 × 10^4^/well RAW264.7 cells and 1 × 10^5^/well BMDMs were inoculated in a 24‐well dish and cultured in a 5% CO_2_ incubator at 37°C for 5 days, separately. Exactly 25 ng/mL MCSF (R & D systems) and 25 ng/mL RANKL (R & D systems) were added to each well, and the solution was refreshed every 2 days.

### 2.2 Cell proliferation assay

RAW264.7 cells and BMDMs (5 × 10^3^ cells per well) were inoculated into 96‐well dishes separately and the experimental groups were divided into five groups. At the same time, different concentrations of PAE (0, 0.1, 1, 10 and 100 mg/mL) were added to continuously culture the cells for 3 days. PAE is a traditional Chinese medicine extracted by Sichuan Good Doctor company through ethanol extraction of Periplaneta americana, mainly including amino acids, polypeptides, nucleosides, and other components. The absorbance was measured using CCK‐8 Kit (Apexbio) at 24 and 72 h, respectively.

### 2.3 TRAP staining

RAW264.7 cells and BMDMs were inoculated in a 24 well culture dish. After the cells adhered to the well, the culture medium was changed and the final concentration of MCSF and RANKL was 25 ng/mL. Meanwhile, different concentrations of PAE (0, 0.1, 1, and 10 mg/mL) were added. The cells were cultured until the fifth day and then stained by a TRAP Staining Kit (Sigma). The TRAP‐positive cells in each well were observed under an inverted microscope and counted; the positive cells were red‐stained and contained at least three nuclei.

### 2.4 Osteoclastic gene detection

The osteoclastic gene expression was detected by RT‐qPCR. 1 × 10^5^ RAW264.7 cells and 1 × 10^6^ BMDMs per well were inoculated into a 6‐well culture dish, respectively. After 5 days of osteoclastic induction, 1 mL Trizol (Invitrogen) was added to each well to lyse the cells. Moreover, the total RNA of the cells was extracted according to the standard process provided by the company. Exactly 1μg of total RNA was retrotranscribed into cDNA according to the instructions of the reverse transcription kit (Takara).^19^, ^20^ Then, the real‐time fluorescence quantitative PCR reaction was conducted according to the instruction of the reagent, which is reacted in Roche Light Cycler® 96 instruments. Afterwards, detection and analysis of osteoclast‐related gene expression were calculated by the 2^−∆∆CT^ method by standardizing with GAPDH gene expression and compared with a control group. The primers used are shown in Table 1.

**TABLE 1:** The primers used in the article

*Gapdh‐*F	ACTGAGGACCAGGTTGTC
*Gapdh‐*R	TGCTGTAGCCGTATTCATTG
*Nfatc1‐*F	GGAGAGTCCGAGAATCGAGAT
*Nfatc1‐*R	TTGCAGCTAGGAAGTACGTCT
*Acp5‐*F	CACTCCCACCCTGAGATTTGT
*Acp5‐*R	CATCGTCTGCACGGTTCTG
*Ctsk‐*F	GAAGAAGACTCACCAGAAGCAG
*Ctsk‐*R	TCCAGGTTATGGGCAGAGATT
*Il1β‐*F	GCAACTGTTCCTGAACTCAACT
*Il1β‐*R	ATCTTTTGGGGTCCGTCAACT
*Il6‐*F	TAGTCCTTCCTACCCCAATTTCC
*Il6‐*R	TTGGTCCTTAGCCACTCCTTC
*Tnfα‐*F	ACGGCATGGATCTCAAAGAC
*Tnfα‐*R	AGATAGCAAATCGGCTGACG

### 2.5 Bone resorption activity test

The extracted healthy human third molar was used to prepare the dentin slices with a diameter of 6 mm and thickness of 100 μm through the hard tissue cutting and grinding machine. After disinfection and sterilization, the slices were placed in a 24‐well culture plate and RAW264.7 cells and BMDMs were inoculated for osteoclastic induction. Meanwhile different concentrations of PAE were added. On the fifth day of cell culture, the dentin slices were taken out, bleached with 10% bleach, and cleaned with an ultrasonic concussion instrument. The slices were observed using an upright metallurgical microscope (Olympus).

### 2.6 Co‐culture of osteoblasts and osteoclasts

The calvaria of mice (6–8 weeks) was digested with type I collagenase (Sigma), filtered with a 70 μm filter, and inoculated into 24‐well culture plate in α‐MEM medium containing 10% FBS. Osteoblasts were obtained after 7 culture days. 1 × 10^5^/well BMDMs were seeded on the osteoblast layer and cultured continuously with 10 nM 1,25‐dihydroxy vitamin D3 (Sigma) and 1 mM prostaglandin E2 (Sigma) for 7 days.^3^ For the co‐culture of 3T3E1 cells and RAW264.7 cells, 5 × 10^4^/well 3T3E1 cells were inoculated into 24‐well plates. After the cells adhered to the well, 1 × 10^4^/well RAW264.7 cells were seeded on the 3T3E1 cell layer. Exactly 10 mM β sodium glycerate (Sigma), 50 μg/mL vitamin C (Sigma), and 100 nM dexamethasone (Sigma) were used to culture the cells continuously for 7 days.

### 2.7 Actin ring staining

After 5 days of osteoclastic induction, Hoechst (Solarbio) and phalloidin‐iFluor 488 Conjugate (Abcam) were used for nuclear staining and actin ring staining. The actin ring staining was observed and counted under an inverted fluorescence microscope (Olympus).

### 2.8 ELISA

RAW264.7 cells and BMDMs were treated with or without PAE in the presence of 100 ng/mL LPS, and the cell culture supernatants were collected to determine the secretory inflammatory cytokine levels according to the manufacturer's instruction.

### 2.9 RNA‐seq

After 3 days of osteoclastic differentiation induction, RNA from untreated and PAE treated RAW264.7 cells was extracted using Trizol reagent. Total amounts and integrity of RNA were assessed using the RNA Nano 6000 Assay Kit of the Bioanalyzer 2100 system (Agilent Technologies, CA, USA). Transcriptome sequencing libraries were prepared using NEBNext® Ultra™ RNA Library Prep Kit for Illumina® according to the manufacture's instruction.^21^ After qualified library inspection, different libraries were pooled according to the requirements of effective concentration and target off‐machine data volume, and sequenced with Illumina NovaSeq 6000. And then, paired‐end clean reads were mapped to Mus Musculus(mm10). Differential expression analysis of two groups (three biological replicates per condition) was performed using the DESeq2 R package (1.20.0). Genes showing |log2(fold‐change)| ≥ 0.5 and *P* value <0.05 were set as the threshold for significantly differential expression.

### 2.10 Western blot

The total protein of cells was extracted through SAB protein lysate (Sab biotechnology). Then, an appropriate amount of concentrated SDS‐PAGE protein loading buffer was added to the collected protein samples and heated in a 100°C metal bath instrument for 5 min to fully denature the protein. Next, gel electrophoresis experiments were initiated using the Omni‐Easy™ ONE‐Step PAGE Gel Fast Preparation Kit (Epizme Biotechnology Co., Ltd) for gel preparation experiments and adding denatured proteins to the gel wells in certain amounts. After that, electrophoretic gel was transferred using PVDF membrane, and at the end of membrane transferring, the protein membrane was immediately placed in the blocking buffer for 1 h.^22^ Then, the blocked protein membranes were incubated with primary antibodies overnight. The following day, after completion of the secondary antibody incubation in the incubation bath, the target protein was detected with a supersensitive ECL luminescent solution. The primary antibodies used in this experiment are as follows: GAPDH (1:100000, Proteintech), p‐STAT1 (1:500, Beyotime Biotechnology), p‐STAT2 (1:500, Beyotime Biotechnology), STAT1 (1:500, Huabio), STAT2 (1:500, Huabio), and TLR3 (1:500, Beyotime Biotechnology).

### 2.11 Statistical analysis

All data were expressed as the mean ± standard deviation of at least three independent experiments. The data were analysed by one‐way ANOVA with Dunnett test or t‐test. A *P* value <0.05 was considered statistically significant.

## 3 RESULTS

### 3.1 Effects of PAE on cell proliferation

**FIGURE 1:**
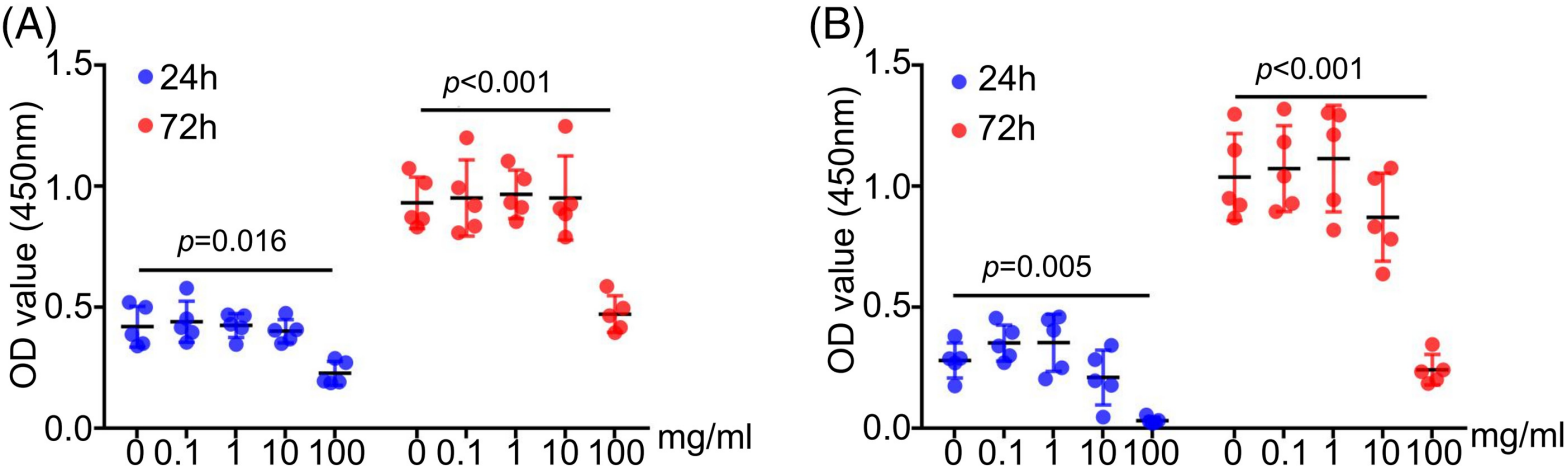
Effects of PAE on cell proliferation. (A) CCK‐8 test of RAW264.7cells on 24 and 72 h after PAE treatment (*n* = 5). (B) CCK‐8 test of BMDMs on 24 and 72 h after PAE treatment (*n* = 5).

To test whether PAE has cytotoxicity or affects cell proliferation, we treated cells with PAE under the concentration of 0, 0.1, 1, 10, and 100 mg/mL, respectively. The CCK‐8 results showed that 100 mg/mL PAE significantly inhibited the proliferation of RAW264.7 cells, and other concentrations of PAE had no significant effect on cell proliferation (Figure 1A). Similar results were observed when exploring the effect of PAE on the proliferation of BMDMs (Figure 1B). These results indicated that PAE had no effect on cytotoxicity or macrophage proliferation unless the concentration was so high. Moreover, based on the results, we used the concentrations of 0.1, 1, and 10 mg/mL in the subsequent experiments.

### 3.2 PAE inhibits osteoclastic differentiation

**FIGURE 2:**
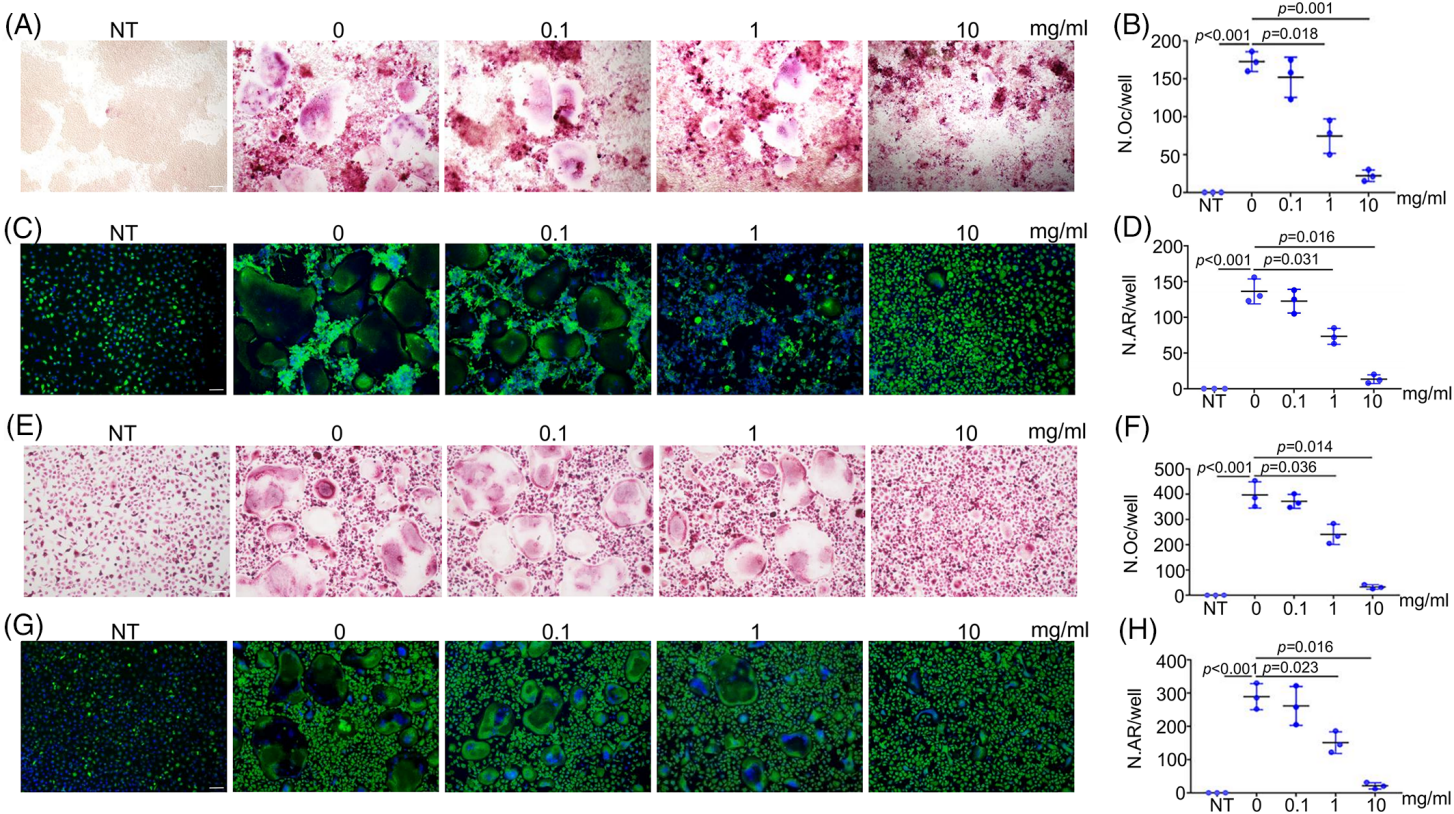
PAE inhibits osteoclastic differentiation. (A,B) TRAP staining and quantification of RAW264.7 cells derived osteoclasts (*n* = 3), scale bar = 100 μm. (C,D) Actin ring staining and quantification of RAW264.7 cells derived osteoclasts (*n* = 3), scale bar = 100 μm. (E,F) TRAP staining and quantification of BMDMs derived osteoclasts (*n* = 3), scale bar = 100 μm. (G,H) Actin ring staining and quantification of BMDMs derived osteoclasts (*n* = 3), scale bar = 100 μm.

We then performed TRAP and actin ring staining after 5 days of osteoclastic induction of RAW264.7 cells. The results showed that 1 mg/mL and 10 mg/mL PAE impaired the osteoclastic differentiation of RAW264.7 cells. Remarkably, in the 10 mg/mL group, the number of TRAP‐positive cells (Figure 2A,B) as well as the number of actin rings (Figure 2C,D) was significantly decreased. As shown in Figure 2E‐H, with the treatment of MCSF and RANKL, 1 mg/mL and 10 mg/mL PAE (especially 10 mg/mL PAE) also suppressed the differentiation of BMDMs into osteoclasts, which were consistent with the results of RAW264.7 cells.

### 3.3 PAE down‐regulates the expression of osteoclastic genes

**FIGURE 3:**
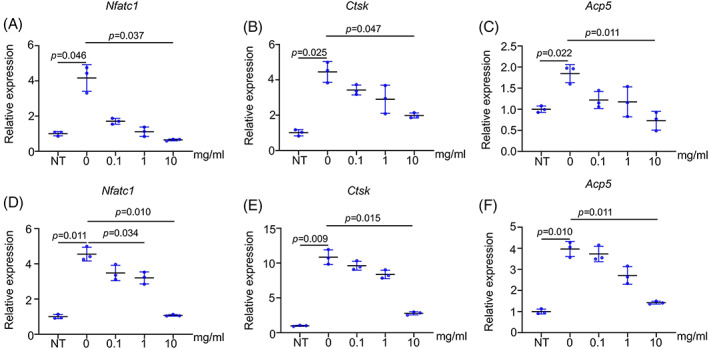
PAE inhibits expression of osteoclastic genes. (A‐C) RT‐qPCR analyses of osteoclastic differentiation‐related genes *Nfatc1*, *Ctsk*, and *Acp5* in RAW264.7 cells after osteoclastic induction (*n* = 3). (D‐F) RT‐qPCR analyses of *Nfatc1*, *Ctsk*, and *Acp5* in BMDMs after osteoclastic induction (*n* = 3).

Next, we performed RT‐qPCR assays to examine the expression levels of *Nfatc1*, cathepsin K (*Ctsk)*, and TRAP (*Acp5)*. The results showed that the concentrations of PAE 10 mg/mL could decrease the expression of *Nfatc1* (Figure 3A), which was also verified in BMDMs (Figure 3D). However, 1 mg/mL PAE also restrained the expression of *Nfatc1 in BMDMs* (Figure 3D). In addition, 10 mg/mL PAE could also significantly inhibit the expression of *Ctsk* and *Acp5* in both RAW264.7cells (Figure 3B,C) and BMDMs (Figure 3E,F).

### 3.4 PAE suppresses osteoclastic resorption

**FIGURE 4:**
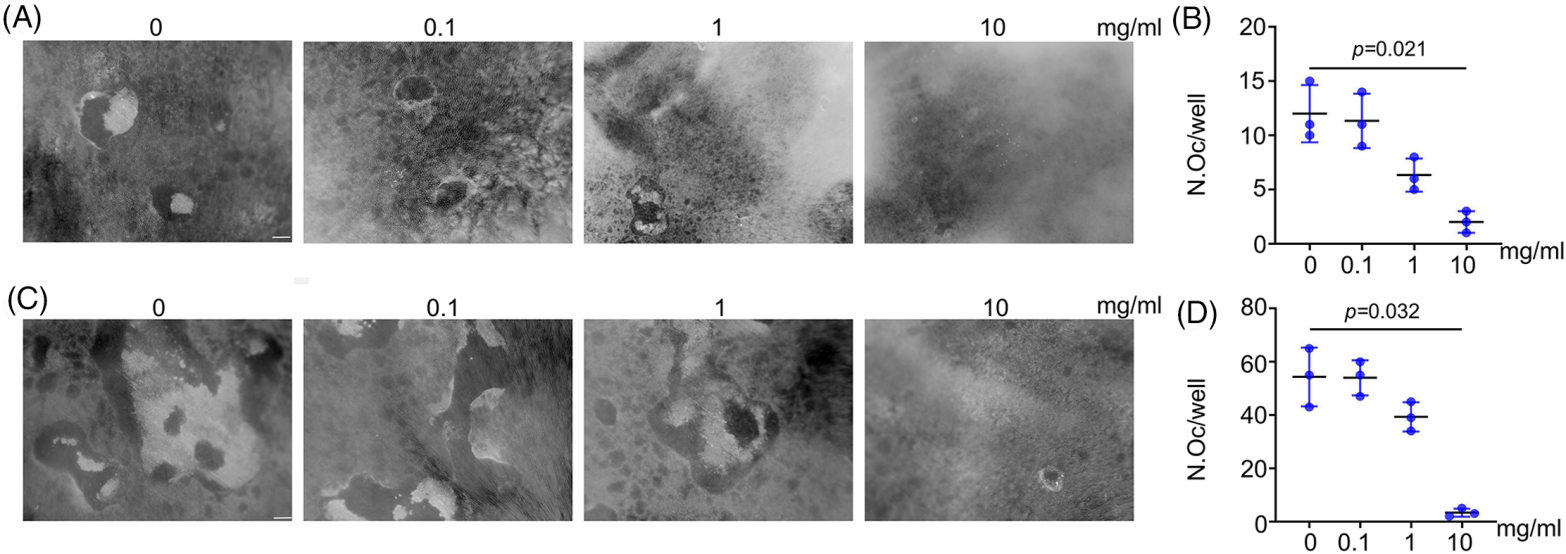
PAE attenuates bone resorption in vitro. (A,B) The area of absorption lacunae and the number of absorption lacunae for RAW264.7 cells derived osteoclasts (*n* = 3), scale bar = 100 μm. (C,D) The area of absorption lacunae and the number of absorption lacunae for BMDMs derived osteoclasts (*n* = 3), scale bar = 100 μm.

To test the capability of bone resorption, RAW264.7 cells were seeded on dentin slices and treated with PAE. Compared with the control group, the area of absorption lacunae and the number of absorption lacunae in PAE (10 mg/mL) treated group decreased (Figure 4A,B). To verify the effect of PAE on the bone resorption function of BMDMs derived osteoclasts, we conducted the same experiment. Our results demonstrated that after osteoclastic differentiation induction, the bone resorption ability of BMDMs was also significantly inhibited by PAE (10 mg/mL) (Figure 4C,D).

### 3.5 PAE inhibits osteoclast formation in a co‐culture system

**FIGURE 5:**
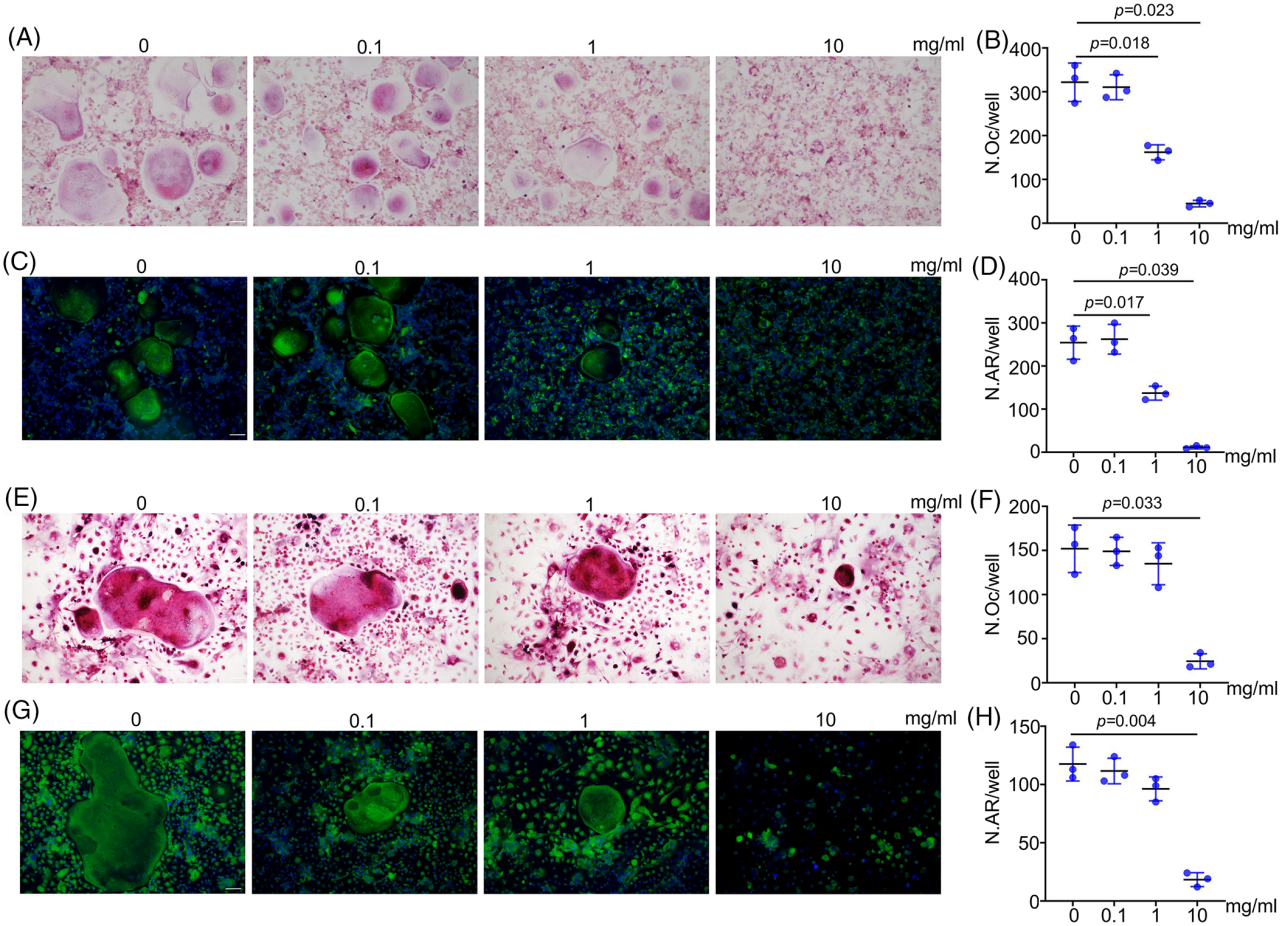
PAE inhibits osteoclast formation in the co‐culture system. (A,B) TRAP staining and quantification of osteoclasts in 3T3E1 and RAW264.7 cells co‐culture system (*n* = 3), scale bar = 100 μm. (C,D) Actin ring staining and quantification of osteoclasts in 3T3E1 and RAW264.7 cells co‐culture system (*n* = 3), scale bar = 100 μm. (E,G) TRAP staining quantification of osteoclasts in calvarial osteoblasts and BMDMs co‐culture system (*n* = 3), scale bar = 100 μm. (F,H) Actin ring staining and quantification of osteoclasts in calvarial osteoblasts and BMDMs co‐culture system (*n* = 3), scale bar = 100 μm.

Next, we co‐cultured 3T3E1 and RAW264.7 cells for 7 days, and observed that both 1 mg/mL and 10 mg/mL PAE significantly reduced the number of TRAP‐positive cells (Figure 5A,B) and actin rings (Figure 5C,D). For the co‐culture of BMMSCs and BMDMs, 10 nM 1,25‐dihydroxy vitamin D3 and 1 mM prostaglandin E2 were added to the α‐MEM medium for 7 days. Consistently, 10 mg/mL PAE reduced the number of osteoclasts (Figure 5E,F) and actin rings (Figure 5G,H).

### 3.6 PAE inhibits LPS‐induced inflammation

**FIGURE 6:**
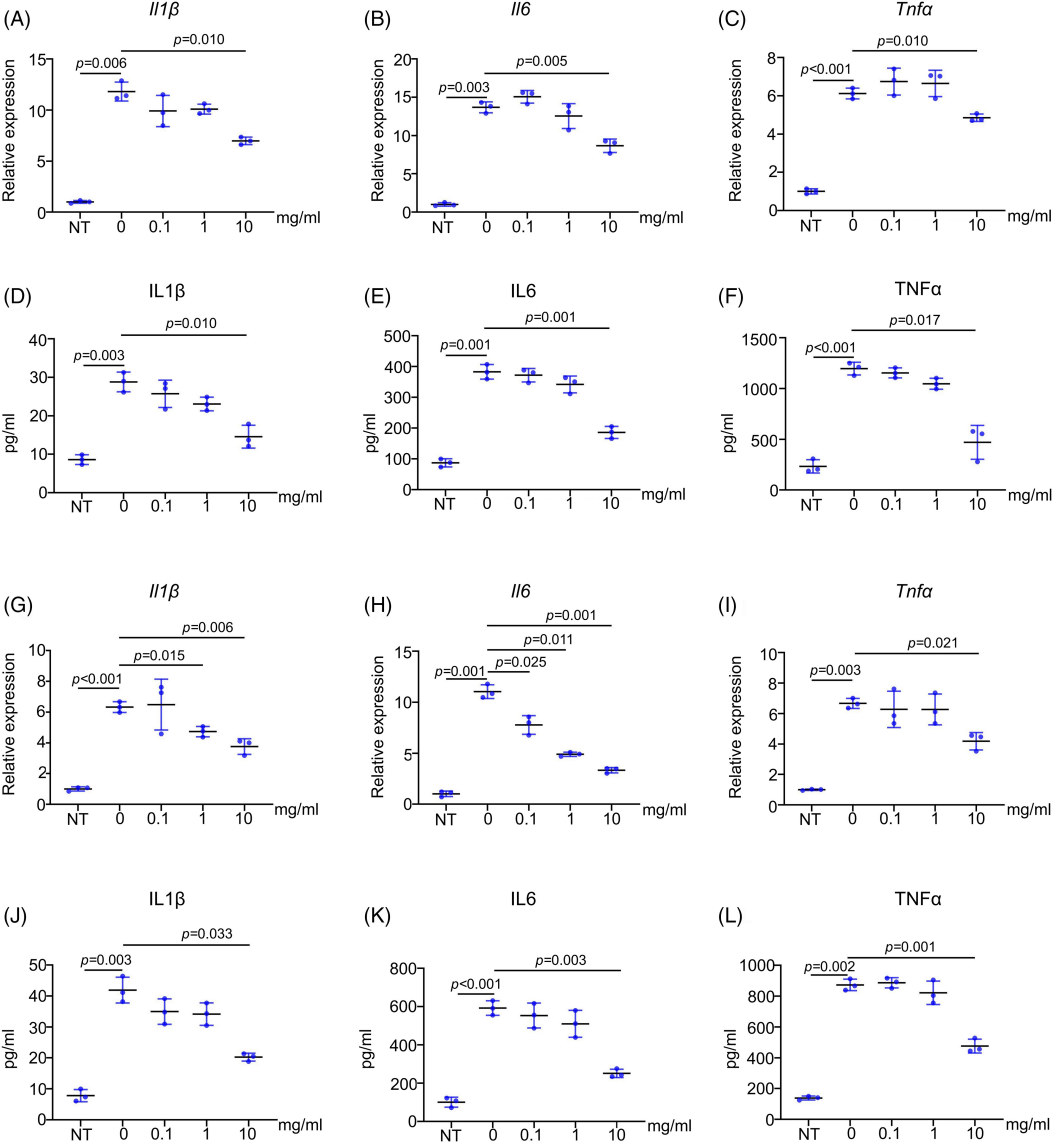
PAE suppresses the LPS‐induced inflammation. (A‐C) RT‐qPCR analyses of *Il1β*, *Il6*, and *Tnfα* in RAW264.7 cells (*n* = 3). (D‐F) ELISA measurements of IL1β, IL6, and TNFα of RAW264.7 cells (*n* = 3). (G‐I) RT‐qPCR analyses of *Il1β*, *Il6*, and *Tnfα* in BMDMs (*n* = 3). (J‐L) ELISA measurements of IL1β, IL6, and TNFα of BMDMs (*n* = 3).

We then treated RAW264.7 cells and BMDMs with 100 ng/mL LPS for 12 h to simulate the inflammatory microenvironment and detected the effect of PAE on inflammatory response in vitro. After PAE treatment, the results showed that LPS could significantly increase the expression level of inflammatory genes *Il1β, Il6*, and *Tnfα* in RAW264.7 cells, and 10 mg/mL PAE effectively reduced the expression levels of these genes (Figure 6A‐C). In addition, the results of ELISA further showed that 10 mg/mL PAE significantly inhibited the secretion of inflammatory factors IL1β, IL6, and TNFα in RAW264.7 cells (Figure 6D‐F). For BMDMs, 10 mg/mL PAE played a similar role (Figure 6G‐L).

### 3.7 PAE coordinates multiple pathways

**FIGURE 7:**
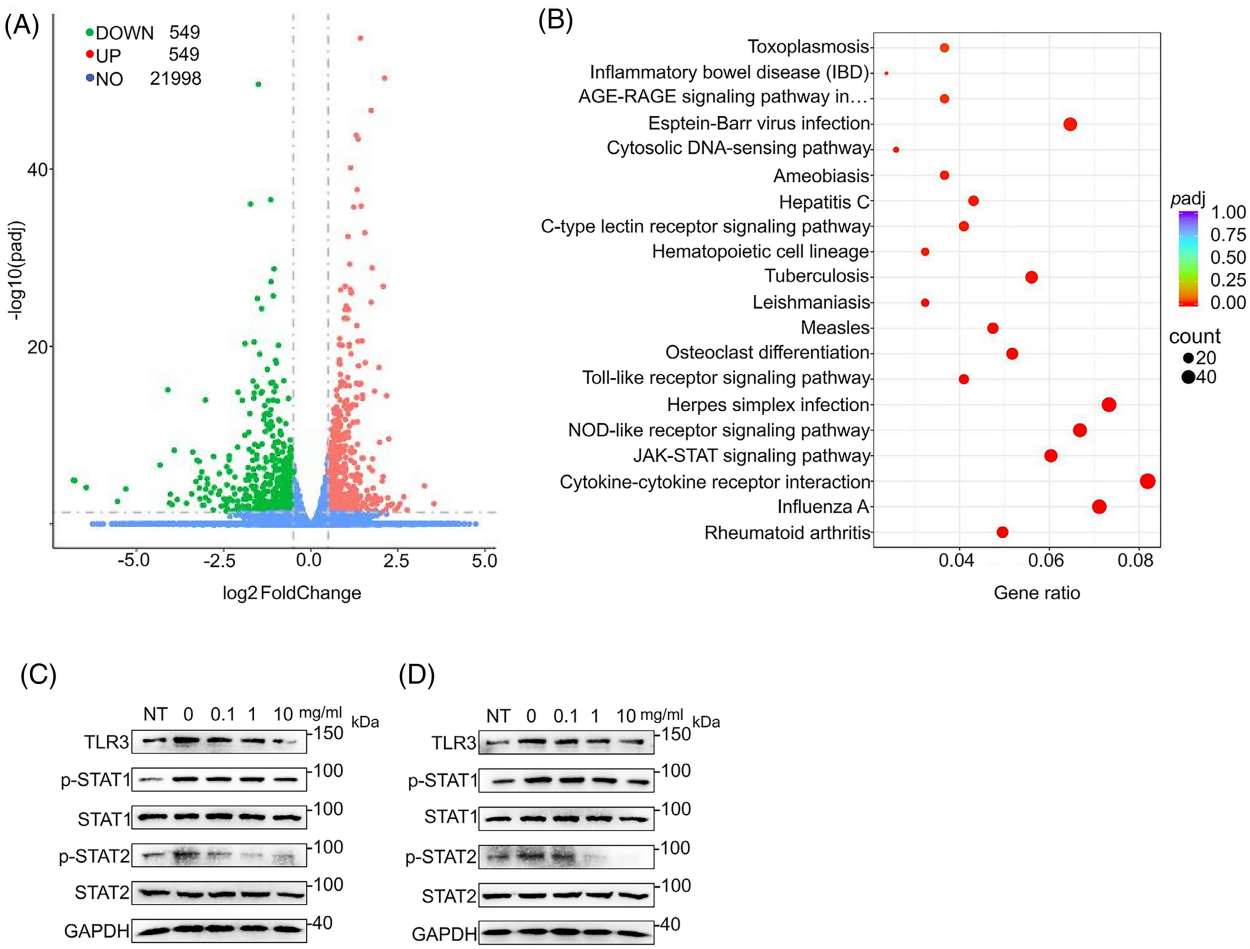
PAE coordinates multiple pathways. (A) Volcano plot of RNA‐seq data from RAW264.7 cells with 3 days of osteoclastic differentiation induction. Up‐regulated genes were labelled in red and down‐regulated genes were labelled in green. (B) KEGG pathway analysis of the top 20 signaling pathways. (C) Western blots of TLR3, p‐STAT1, p‐STAT2, STAT1, and STAT2 in RANKL treated RAW264.7 cells for 30 min. (D) Western blots of TLR3, p‐STAT1, p‐STAT2, STAT1, and STAT2 in RANKL treated BMDMs for 30 min.

To dissect the mechanism, we extracted RNA from RAW264.7 cells with or without PAE treatment and performed RNA sequencing analysis. Among the 21,998 candidate genes, 549 were up‐regulated and 549 were down‐regulated after PAE treatment (Figure 7A). KEGG enrichment analysis showed that JAK–STAT, NOD‐like receptor signaling pathway, and Toll‐like receptor signaling pathway were enriched (Figure 7B).

To validate this observation, we conducted western blot analysis and found that PAE decreased the phosphorylation of STAT1 and STAT2, both of which were key factors in NOD‐like receptor and JAK–STAT signalling pathway (Figure 7C,D). In addition, PAE significantly inhibited the activation of TLR3, a key factor in Toll‐like receptor signalling pathway (Figure 7C,D).

## 4 DISCUSSION

In the development of osteoporosis, osteoclasts play a major role in bone resorption. The progress rate and duration of bone resorption depend on the bone resorption activity of osteoclasts and the number of osteoclasts.^23^ If the bone resorption activity of osteoclasts increases, the range of bone resorption increases. On the contrary, the absorption range decreases. Inhibiting the cell viability, osteoclastic differentiation, and inflammation could be used as a potential strategy for the treatment of osteoporosis.

Osteoclasts are multinucleated giant cells formed by the fusion of mononuclear macrophages differentiated from myeloid progenitor cells in the bone marrow. The early immature proliferative mononuclear phagocytes are called osteoclast precursors, which fuse into multinucleated cells and activate into osteoclasts under the stimulation of various signal factors.^24^ BMDMs and macrophage cell line RAW264.7 are two osteoclast precursor cell models.^25^, ^26^ Therefore, in our study, we used these two cells to study the effect of PAE on its osteoclastic differentiation. However, it is a crucial aspect to examine the influence of PAE on cytotoxicity and cell proliferation when assessing the properties of PAE. For BEL‐7402, the hepatocellular carcinoma cell line, and BEL‐7402/5‐FU (BEL‐7402 with multidrug resistance), extracts from Periplaneta americana suppressed their proliferation at high dose but showed no effects when the concentration was not too high.^27^ This result was similar to our experiment in that PAE affected the proliferation of both RAW264.7 cells and BMDMs in a dose‐dependent manner, and low‐dose PAE did not change the proliferation state of macrophages. However, Zou et al. reported that PAE promoted the proliferation of hepatocytes at low doses.^28^ In addition, PAE was also shown to accelerate the proliferation of keratinocytes^29^ and fibroblasts.^30^ We speculate that a high concentration of PAE presents toxicity to cells. However, when the concentration is equal to or low than 1 mg/mL, whether PAE enhances cell proliferation may depend on the type of cells.

Emerging evidence has suggested that traditional Chinese medicine can attenuate bone resorption under circumstances including osteoporosis. As mentioned above, Chinese herbal medicine, such as flavonoids and saponins, have been proven to function in anti‐resorptive activities. However, the related herbal ingredients or formulas are complex and more effective and safer. PAE is a traditional Chinese medicine that is deeply researched animal medicine and has a modern preparation form. Kangfuxin Liquid and Xinmailong Injection are extracted from Periplaneta americana and are highly recognized for their efficacy in treating wound healing, kidney and cardiovascular disease, etc.^14^, ^31^ Interestingly, in our study, we found that PAE had inhibitory effects on the differentiation of osteoclasts, including inhibiting the formation of osteoclasts, down‐regulating the expression of osteoclast genes, and reducing the activity and function of osteoclasts, indicating its potential as an alternative strategy for the treatment of osteoporosis.

Inflammation may appear as a concomitant symptom of osteoporosis. Therefore, reducing the secretion of inflammatory factors and the expression of inflammatory genes is an effective therapeutic strategy. IL1β, IL6, and TNFα are important inflammation‐related factors, which have been reported in many research studies.^32^, ^33^, ^34^ In our study, we demonstrated that 10 mg/mL PAE could obviously inhibit the expression and secretion of IL1β, IL6, and TNFα. The findings would contribute to the application of PAE to reduce inflammatory response.

Osteoclastic differentiation is a sophisticated process regulated by various molecules and signaling pathways. Synthesized medicines are usually designed to target specific mediators during osteoclastogenesis. For instance, denosumab is a human monoclonal antibody against RANKL, essential for osteoclast formation and function.^35^ Another example is Odanacatib, an inhibitor of cathepsin K. Cathepsin K secreted by osteoclasts is responsible for type I collagen degradation in bone tissue, thus the administration of Odanacatib leads to the abrogation of bone resorption.^36^ Concerning PAE, it contains at least 12 chemical components.^37^ Thus, PAE may have multiple targets to prevent osteoclastogenesis, which our RT‐qPCR results and RNA‐seq results have partly demonstrated. Comprehensively, the genes of *Nfatc1*, *Ctsk*, and *Acp5* and the signaling pathways of JAK–STAT, NOD‐like receptor signaling pathway, and Toll‐like receptor signaling pathway were targeted by PAE. This means that, on the one hand, PAE is potent to control osteoclastic differentiation through comprehensive mechanisms. However, on the other hand, much work has to be done to verify the safety and efficacy of PAE before its application in osteoporosis.

Collectively, PAE could effectively suppress osteoclastic differentiation, formation, and bone resorption. Potential mechanisms assisting PAE with the inhibitive effects on osteoclastic differentiation and function have also been revealed via RNA‐seq analysis. PAE may provide a potential pharmacological strategy for osteoporotic bone disease as traditional Chinese medicine.

## AUTHOR CONTRIBUTIONS

The experiments were conceived and designed by Quan Yuan and Chenchen Zhou and carried out by Bin Zhao and Yuning Zhang. Jie Xu and Yuyu Li assisted in carrying out the experiments. The data were analysed by Bin Zhao, Yuning Zhang, and Jie Xu. Moreover, the article was written by Bin Zhao and Yuyu Li and revised by Quan Yuan and Chenchen Zhou. The article was read and approved by all the authors.

## CONFLICT OF INTEREST

All authors declare that there is no conflict of interest.

## Data Availability

Data supporting the results of this study can be obtained from the corresponding author upon reasonable request.
